# Development and internal validation of a monocyte-to-albumin ratio-based risk assessment model for coronary artery lesions in Kawasaki disease

**DOI:** 10.3389/fped.2026.1881684

**Published:** 2026-08-18

**Authors:** Min-Min Li, Gao-Min Liu

**Affiliations:** 1 Meizhou Clinical Medical College of Shantou University Medical College, Meizhou, China; 2 Department of Pediatrics, Meizhou People’s Hospital, Meizhou, China; 3 Department of Hepatobiliary Surgery, Meizhou People’s Hospital, Meizhou City, China

**Keywords:** coronary artery lesions, kawasaki disease, machine learning, mar, nomogram

## Abstract

**Introduction:**

Coronary artery lesions (CAL) are the most severe complication of Kawasaki disease (KD), yet accurate and practical risk assessment tools remain limited. This study aimed to develop and validate a parsimonious logistic regression model for CAL risk assessment using routine clinical and laboratory parameters.

**Methods:**

A total of 324 consecutive KD patients were retrospectively enrolled. Candidate predictors were screened by univariate analysis and a strict consensus-based strategy integrating five feature-selection methods (LASSO, random forest, Boruta, recursive feature elimination, and univariate threshold). Ten machine learning algorithms were compared, and the final model was selected based on performance and interpretability. A nomogram, calibration curve, and an online calculator were developed.

**Results:**

Among 324 patients, 64 (19.8%) had baseline CAL. Four variables [fever duration, GGT, monocyte-to-albumin ratio (MAR), and ESR] were consistently selected by all five methods. Logistic regression yielded a Brier score of 0.142, with high specificity (0.846), but limited sensitivity (0.421) on the hold-out test set. No significant effect modification was observed in exploratory interaction analyses assessing the association of MAR on CAL across the evaluated covariates (all adjusted *P* > 0.05). GBM model offered superior sensitivity (0.684) than other models, suggesting a potential exploratory role in sensitivity-driven screening scenarios. An online calculator was deployed strictly for exploratory research purposes.

**Conclusions:**

A simple four-variable logistic model incorporating fever duration, GGT, ESR, and MAR provides preliminary CAL risk assessment. However, the modest event count and single-center nature mandate multi-center external validation before any clinical application.

## 1 Introduction

Kawasaki disease (KD) is an acute, self-limited systemic vasculitis that predominantly affects infants and young children. Although the etiology remains elusive, the most serious complication is the development of coronary artery lesions (CAL), which can lead to long-term cardiovascular sequelae including myocardial infarction, ischemic heart disease, and even sudden death (1, 2). Even with timely intravenous immunoglobulin (IVIG) therapy, approximately 15%–20% of children still develop CAL, highlighting the urgent need for accurate early risk assessment (3, 4). Current clinical scoring systems have limited assessement performance, and many rely on complex or region-specific variables, making them less generalizable (5). Therefore, identifying simple, widely available biomarkers for early identification of CAL remains a priority.

Routine blood parameters have gained increasing attention in KD research due to their low cost, rapid availability, and ability to reflect systemic inflammation and nutritional status. Several studies have reported associations between CAL and conventional markers such as C-reactive protein, platelet count, hemoglobin, and albumin (6–8). More recently, composite inflammatory indices derived from blood cell counts and biochemical measures, such as neutrophil-to-lymphocyte ratio (NLR), platelet-to-lymphocyte ratio (PLR), and systemic immune-inflammation index (SII), C-reactive protein-albumin-lymphocyte (CALLY) (9, 10)- have been explored for their clinical associations in KD (8, 9). The monocyte-to-albumin ratio (MAR) is a novel index that integrates monocyte count (a marker of innate immune activation) and albumin (a negative acute-phase protein reflecting nutritional and inflammatory status) (11, 12). To date, however, no study has investigated the role of MAR in identifying CAL in KD patients, and the potential of MAR to serve as an innovative and practical biomarker remains unexplored.

In this study, we aimed to develop and validate a parsimonious logistic regression model incorporating MAR along with other routine clinical and laboratory parameters for early identification of CAL in children with KD. We further compared ten machine learning algorithms, selected the optimal model based on performance and interpretability, and constructed an online risk calculator to facilitate exploratory use.

## 2 Materials and methods

### 2.1 Study population and data collection

This retrospective study included consecutive children diagnosed with KD at Meizhou People's Hospital from January 2020 to January 2026. The diagnosis of KD was based on the American Heart Association 2017 criteria (1). Inclusion criteria were: (1) first episode of KD; (2) complete clinical and laboratory data within 7 days before intravenous immunoglobulin (IVIG) treatment; (3) echocardiographic evaluation performed at baseline (within 7 days before IVIG treatment) and at follow-up. Exclusion criteria were: (1) recurrent KD; (2) history of congenital heart disease or other inflammatory diseases; (3) incomplete data on key variables (including fever duration, GGT, monocyte count, albumin). A total of 324 patients met the criteria and were enrolled. The study was approved by the Ethics Committee of Meizhou People's Hospital (approval no. 22022-C-71).

The following demographic, clinical, and laboratory data were extracted from electronic medical records: age, sex, fever duration (days, before admission), white blood cell count (WBC), neutrophil count (NEUT), lymphocyte absolute count (LYMPH, ×10^9^/L), monocyte absolute count (MONO, ×10^9^/L), platelet count (PLT, ×10^9^/L), hemoglobin (HGB, g/L), albumin (ALB, g/L), alanine aminotransferase (ALT, U/L), aspartate aminotransferase (AST, U/L), gamma-glutamyl transferase (GGT, U/L), total bilirubin (TBil, µmol/L), direct bilirubin (DBil, µmol/L), creatine kinase (CK, µmol/L), lactate dehydrogenase (LDH, U/L), fibrinogen (Fib, g/L), C-reactive protein (CRP, mg/L), procalcitonin (PCT, ng/mL), erythrocyte sedimentation rate (ESR, mm/h), and the derived index MAR (monocyte-to-albumin ratio × 100, calculated as MONO/ALB × 100). All laboratory specimens were collected before IVIG or aspirin administration. Notably, patients who subsequently developed IVIG resistance were not excluded from this study. The primary objective was to evaluate early predictors measurable prior to IVIG treatment; thus, IVIG resistance was not used as an eligibility criterion.

### 2.2 Outcome definition

The primary outcome was baseline coronary artery lesion (CAL), defined according to the 2017 AHA guidelines (1): (1) left anterior descending or right coronary artery *Z*-score ≥2.5; (2) presence of coronary artery aneurysm (diameter ≥3 mm in children <5 years or ≥4 mm in children ≥5 years). Baseline echocardiograms were obtained within 7 days of illness onset prior to IVIG treatment and were reviewed by two experienced cardiologists blinded to clinical data. Therefore, the assessment models developed in this study are designed to estimate the risk of CAL present at the time of baseline assessment (i.e., pre-treatment CAL). Notably, although follow-up echocardiograms were obtained during the subacute phase to monitor for the persistence or resolution of coronary abnormalities, aneurysms as persistent vs. transient was not classified in this study. The primary outcome and the assessment target for all developed models were strictly limited to the binary baseline CAL status (presence vs. absence). Therefore, the criteria for differentiating persistent and transient aneurysms during follow-up were not incorporated into our modeling framework. Patients were classified into CAL group or non-CAL group based solely on the baseline echocardiography findings.

### 2.3 Data preprocessing and variable selection

The complete dataset was first randomly partitioned into training and hold-out internal test sets using createDataPartition in R (caret package) (13) with stratification by CAL status and a fixed random seed (512) to ensure reproducibility. Missing values (missing proportions ranging from 0.6% to 8.3%, as detailed in Table 1) were then handled separately: the training set was imputed using multivariate imputation by chained equations (MICE) with 5 imputations (14). The imputation model included all demographic and laboratory candidate variables, as well as the outcome variable (CAL), to preserve the underlying relationships among the predictors and the outcome. To facilitate a transparent and reproducible downstream workflow, the results from the five imputed datasets were pooled into a single final imputed training dataset by taking the median of the imputed values across the five iterations. All subsequent steps-including univariate screening, the five feature selection methods, logistic regression model fitting, 10-fold repeated cross-validation, and bootstrap resampling-were performed once on this single pooled dataset, rather than being repeated across each of the five imputed datasets. The hold-out internal test set was imputed using the medians of the imputed training set (or the MICE-derived imputation models) to ensure no information from the test set influenced the training process. The training set was used for feature selection and model development. The hold-out test set was used only for descriptive performance benchmarking and was not involved in model training or tuning. The optimal cut-off value was determined in the training set using the Youden index. This fixed threshold was then applied to the independent test set to evaluate the model's real-world fixed-decision utility. Importantly, all validation procedures described here are internal, and no external validation was performed in this study.

**Table 1:** Baseline characteristics of the included patients with Kawasaki disease.

Variables	Level	No CAL (*n* = 260)	CAL (*n* = 64)	*P* value
Sex (%)	Male	168 (64.6)	42 (65.6)	1.000
	Female	92 (35.4)	22 (34.4)	
Age (months)		24.00 [12.00, 48.00]	24.00 [11.00, 48.00]	0.289
Fever duration (days)		5.00 [4.00, 6.00]	5.50 [4.00, 8.00]	**<0**.**001**
WBC count (*10^9^/L)		15.05 [11.62, 19.02]	16.45 [12.02, 19.18]	0.304
NEUT count (*10^9^/L)		72.70 [60.48, 81.15]	68.90 [58.68, 80.45]	0.243
LYMPH count (*10^9^/L)		2.67 [1.70, 4.31]	3.32 [1.85, 5.31]	0.161
PLT (*10^9^/L)		327.00 [272.00, 409.00]	356.00 [275.50, 449.25]	0.337
HGB (g/L)		114.00 [106.00, 123.00]	113.50 [99.75, 122.25]	0.238
ALB (g/L) (missing: 1.2%)		35.60 [32.90, 38.20]	34.50 [32.00, 37.00]	0.109
GGT (U/L) (missing: 0.6%)		47.00 [17.00, 145.00]	68.00 [31.00, 197.50]	**0**.**043**
CK (U/L) (missing: 1.2%)		51.00 [34.00, 77.00]	38.00 [27.00, 70.50]	**0**.**028**
LDH (U/L) (missing: 1.2%)		302.00 [253.00, 357.00]	303.00 [268.00, 342.50]	0.589
Fib (g/L) (missing: 8.3%)		5.94 [5.25, 6.80]	6.25 [5.00, 7.06]	0.302
ESR (mm/h) (missing: 3.4%)		49.00 [35.00, 62.00]	46.50 [33.25, 55.00]	**0**.**093**
PCT (ng/mL) (missing: 0.6%)		0.84 [0.35, 2.38]	0.64 [0.26, 1.64]	0.184
MAR (missing: 1.2%)		2.49 [1.70, 3.64]	3.27 [1.94, 4.45]	**0**.**013**

CAL, coronary artery lesions; WBC, white blood cell count; NEUT, absolute neutrophil count; LYMPH, absolute lymphocyte count; PLT, platelet count; HGB, hemoglobin; ALB, albumin; GGT, gamma-glutamyl transferase; CK, creatine kinase; LDH, lactate dehydrogenase; Fib, fibrinogen; ESR, erythrocyte sedimentation rate; PCT, procalcitonin; MAR, monocyte-to-albumin ratio (calculated as monocyte count/albumin × 100).

Missing data rates are presented as numbers (percentage) next to the corresponding variables where applicable.

Bold *P* value: *P* < 0.10.

To identify robust predictors, we first performed univariate logistic regression for each candidate variable (variables including demographics, laboratory parameters, and MAR) on the training set. Variables with *p* < 0.05 were considered potential predictors. Then five feature selection methods were applied: 1) Univariate logistic regression *p*-value threshold (*p* < 0.05). 2) LASSO regression (15) (glmnet, alpha = 1) with 10-fold cross-validation; variables with non-zero coefficients at lambda.min were selected. 3) Random forest (16) (randomForest, ntree = 500); top 10 variables by mean decrease Gini. 4) Boruta algorithm (Boruta) (17), a random-forest wrapper that compares original features with shadow features; confirmed attributes (with or without tentative) were selected. 5) Recursive feature elimination (RFE) using random forest as the learner (caret::rfe) (13), with 10-fold cross-validation and subset sizes from 1 to 5; the final set was the top predictors from the best subset by accuracy. To integrate the results, a strict consensus strategy was adopted: only variable chose by all five feature selection methods were considered as candidates for the final model. Moreover, we assessed multicollinearity using variance inflation factor (VIF) and planned to exclude variables with VIF > 5 to avoid instability.

No formal *a priori* sample size calculation was performed given the retrospective and exploratory nature of this study. For the final parsimonious logistic regression model containing four predictors (fever duration, GGT, ESR, and MAR), the number of CAL events in the training set (*n* = 45) yielded an events-per-variable (EPV) ratio of 11.25 (45/4), which exceeds the widely recommended minimum threshold of 10 for reliable logistic regression coefficient estimation and avoids overfitting (18). Although this ratio meets the widely recommended minimum threshold of 10, the modest training set size is acknowledged to inherently carry a risk of overfitting. Notably, the feature selection stage initially considered 16 candidate variables, and the subsequent comparison of ten machine learning algorithms was primarily exploratory.

### 2.4 Logistic regression model development and nomogram

Multivariable logistic regression was performed to construct the logistic regression model based on the consistently selected variables identified above. The logistic regression equation was derived from the coefficients estimated on the training set. Based on this model, a nomogram was constructed using the rms package to facilitate individualized risk estimation.

Three levels of performance evaluation were explicitly distinguished: (1) apparent performance on the training set; (2) internal validation via 10-fold repeated cross-validation (repeats = 5) and bootstrap resampling (200 replicates) to obtain optimism-corrected, bias-adjusted estimates-these serve as our primary internal validation metrics; (3) split-sample internal validation and Firth penalized logistic regression [to address potential small sample bias (19)] on the hold-out test set, which provides descriptive performance but should be interpreted cautiously given its limited sample size.

Additionally, restricted cubic spline (RCS) regression with three knots was employed to examine the dose-response relationship between MAR and CAL, adjusting for other independent predictors. To explore whether the identifying effect of MAR on CAL was modified by other clinical or laboratory variables, interaction analyses were used by adding product terms (MAR × covariate) to the multivariable logistic regression models. To preserve statistical power and avoid information loss inherent in dichotomization, all continuous covariates were kept as continuous variables in these interaction tests. The likelihood ratio test was used to compare models with and without the interaction term. Given the limited number of CAL events and the exploratory nature of these analyses, the interaction *P*-values were adjusted using Bonferroni correction for multiple testing, and should be interpreted descriptively as hypothesis-generating only.

### 2.5 Exploratory machine learning models and SHAP interpretability

To explore whether complex models could improve sensitivity for early screening, ten machine learning algorithms were implemented using the training set: Logistic Regression (glm), Support Vector Machine with radial kernel (svmRadial), Gradient Boosting Machine (gbm), Neural Network (nnet), Random Forest (extraTrees), XGBoost (xgbTree) (20), k-Nearest Neighbors (knn), AdaBoost (AdaBoost.M1), LightGBM (lightgbm) (21), and CatBoost (catboost) (22). All models were trained using 10-fold repeated cross-validation (repeats = 5) with class probabilities enabled and the area under the ROC curve (AUC) as the tuning metric. Hyperparameters were optimized using predefined grids. To address the risk of overfitting, all complex models were trained under the strict framework of 10-fold repeated cross-validation (repeats = 5) with AUC as the optimization metric, and hyperparameters were selected from predefined grids designed to favor model parsimony where possible. Nevertheless, given the modest sample size and the inherently high model flexibility of tree-based ensemble algorithms (e.g., Random Forest and LightGBM), perfect training-set performance was considered a warning sign of model over-parametrization rather than a reliable indicator of clinical generalization. Therefore, these complex models were treated strictly as exploratory benchmarks to evaluate the robustness of simpler alternatives, and their standalone clinical utility was deliberately deprioritized in this study. For each model, the optimal probability threshold was determined on the training set by maximizing the Youden index, and this fixed threshold was applied to the hold-out test set for a fair real-world comparison. Discrimination, calibration, and clinical utility of all machine learning models were assessed using ROC curves, calibration curves (with 200 bootstrap resamples for optimism correction), and decision curve analysis (DCA) (23), respectively. The best model was selected based on the best trade-off between model performance and interpretability.

To interpret the “black-box” performence from complex models, particularly for GBM, the SHAP (SHapley Additive exPlanations) method was applied. This approach provided global feature importance rankings across all predictors and generated local waterfall plots to visualize the contribution of each variable to individual patient risk assessments, thereby enhancing the clinical transparency of the machine learning outputs.

### 2.6 Online dynamic tool for exploratory use

To facilitate exploratory use by researchers and clinicians, a web-based dynamic calculator was developed using the Shiny package in R and deployed it on shinyapps.io. To prevent premature clinical application, a prominent disclaimer is displayed on the interface, stating that the tool is based on a single-center internal hold-out test and is for research purposes only, requiring external validation before any clinical use.

### 2.7 Statistical software and data reporting

All statistical analyses were performed using R software (version 4.5.1). The following packages were used: tidyverse, caret, glmnet, randomForest, Boruta, rms, pROC, DynNom, shapviz, and shiny. Due to non-normal distribution, continuous variables are reported as median [interquartile range] and categorical variables as numbers (percentages). Group comparisons between CAL and non-CAL patients employed the Mann–Whitney *U*-test for continuous variables and the Chi-square or Fisher's exact test for categorical variables. A two-sided *P*-value <0.05 was considered statistically significant.

## 3 Result

### 3.1 Baseline characteristics of the study population

**Figure 1:**
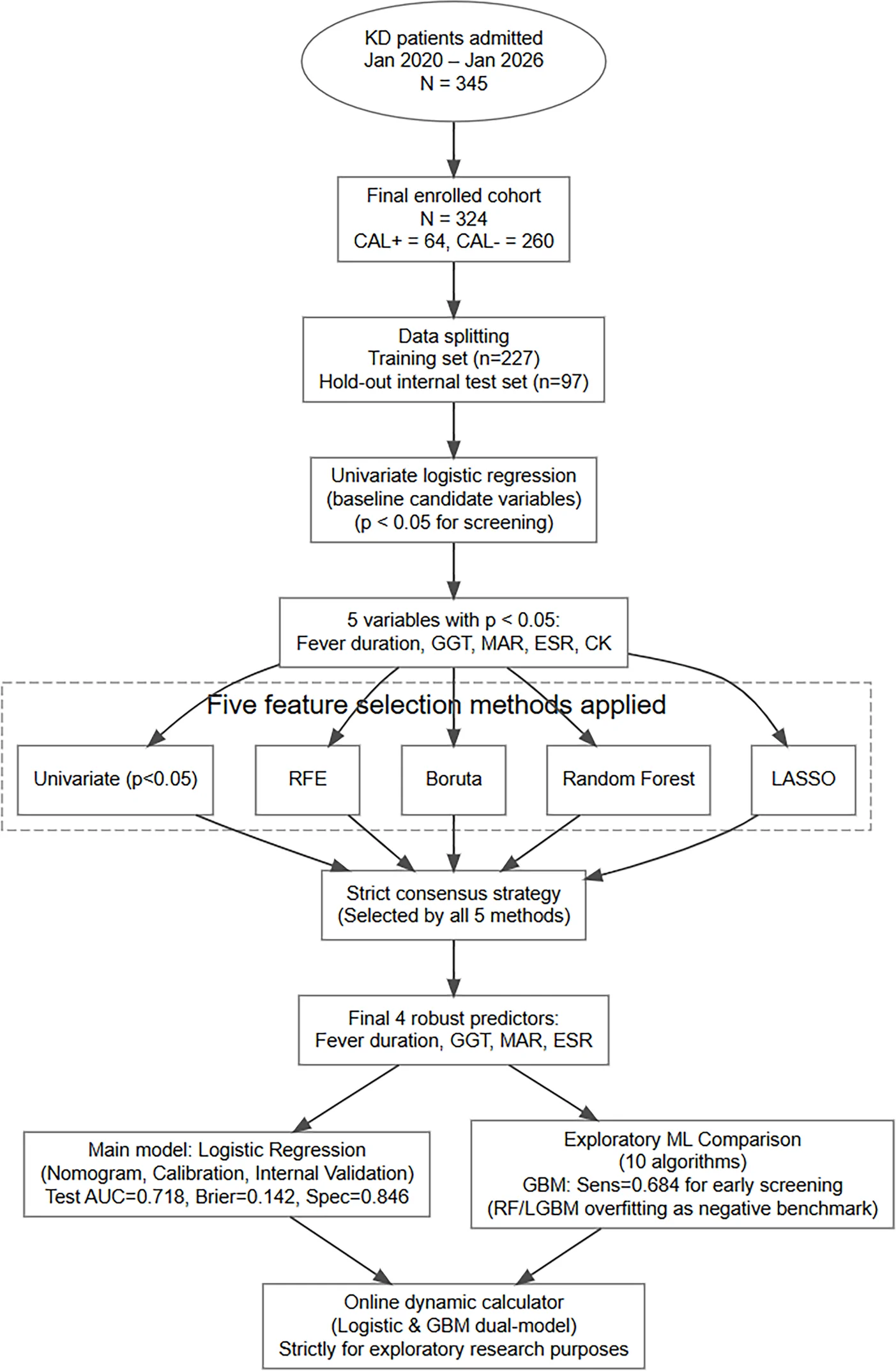
Study flow diagram. KD, Kawasaki disease; CAL, coronary artery lesions; GGT, gamma-glutamyl transferase; MAR, monocyte-to-albumin ratio; ESR, erythrocyte sedimentation rate; CK, creatine kinase; RFE, recursive feature elimination; ML, machine learning; SVM, support vector machine; GBM, gradient boosting machine; NN, neural network; RF, random forest; XGBoost, extreme gradient boosting; KNN, k-nearest neighbors; DCA, decision curve analysis; RCS, restricted cubic spline.

The study flow was showed in Figure 1. A total of 324 consecutive children with KD were enrolled, including 260 (80.2%) without CAL (non-CAL) and 64 (19.8%) had baseline CAL. Baseline demographic, clinical, and laboratory characteristics of the two groups are summarized in Table 1. Compared with the non-CAL group, CAL patients had significantly longer fever duration (median 5.50 vs. 5.00 days, *P* < 0.001) elevated levels of GGT (median 68.00 vs. 47.00 U/L, *P* = 0.043), and larger MAR (median 3.27 vs. 2.49, *P* = 0.013). No statistically significant differences were observed for age, sex, white blood cell count, neutrophil count, lymphocyte count, platelet count, HGB, albumin, LDH, fibrinogen, ESR, or PCT between the two groups.

### 3.2 Feature selection and identification of predictors

**Figure 2:**
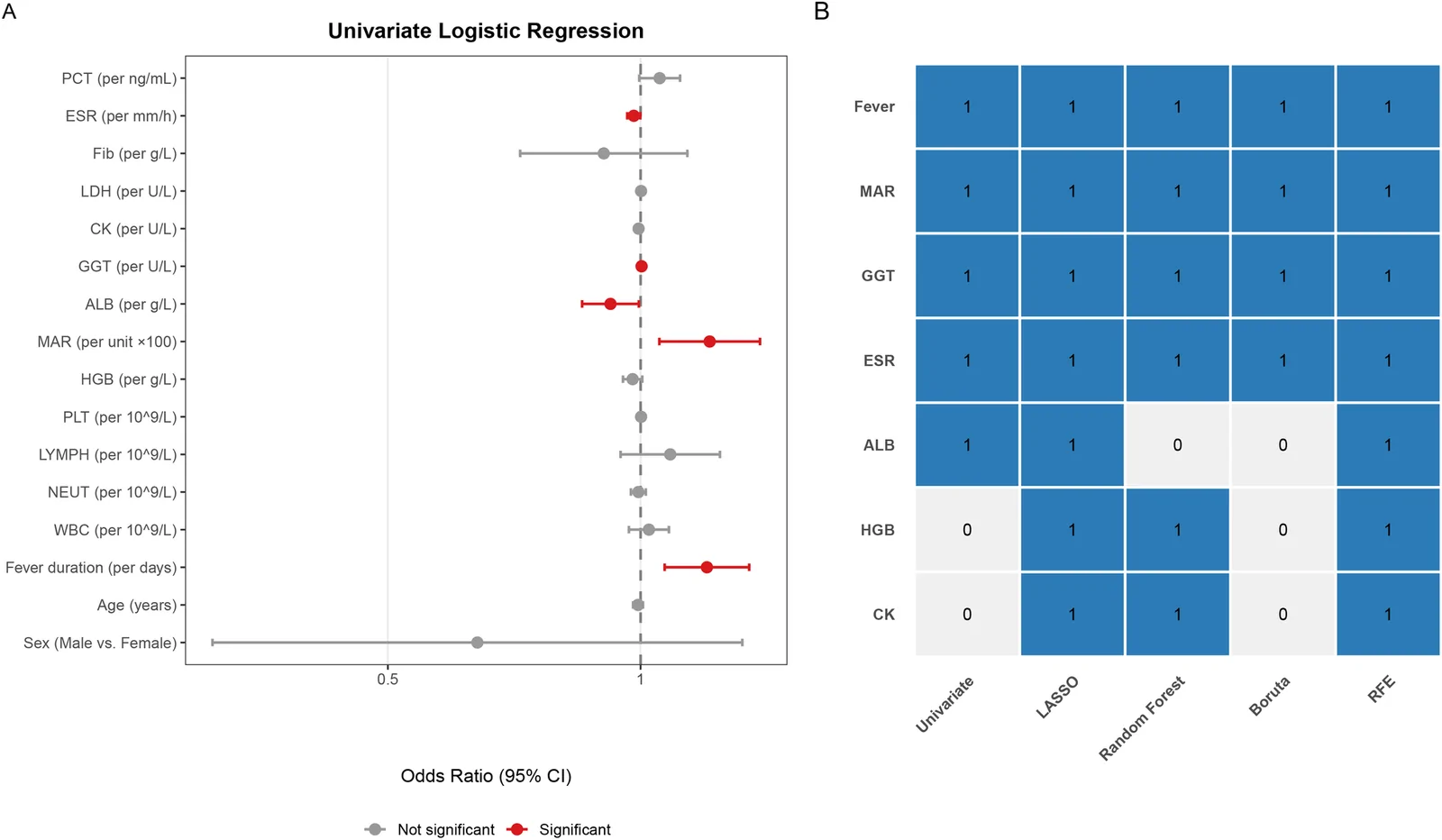
Univariable logistic regression forest plot and and feature selection matrix based on a strict consensus strategy. **(A)** Forest plot of univariable logistic regression for each candidate variable. Odds ratios (ORs) and 95% confidence intervals are plotted on a log scale. Red points and error bars indicate variables with *P* < 0.05; gray indicates non-significant variables. The vertical dashed line represents OR = 1. **(B)** Feature selection heatmap illustrating the intermediate selection status of the evaluated variables across five independent methods (Univariate *p*-value threshold, LASSO, Random Forest, Boruta, and Recursive Feature Elimination). Blue cells indicate selection by the corresponding method; grey cells indicate non-selection. Only variables selected by all five methods were retained as candidates for the final model.

Patients were classified into the training set (*n* = 227) and evaluated on the hold-out internal test set (*n* = 97). No significant differences in baseline characteristics were observed between the two groups (Supplementary Table S1). Univariate logistic regression identified five variables with *P* < 0.05: fever duration, GGT, MAR, ESR, and CK (Figure 2A). To further refine variable selection, five feature selection methods were then applied to the training set (*n* = 227): univariate *p*-value threshold, LASSO, random forest importance, Boruta, and recursive feature elimination (RFE). As showed in Figure 2B, four variables including fever duration, GGT, MAR, and ESR were consistently selected by all five methods. Therefore, these four variables were included in the subsequent multivariable analysis.

### 3.3 Logistic regression model and internal validation

#### 3.3.1 Model specification and nomogram

**Figure 3:**
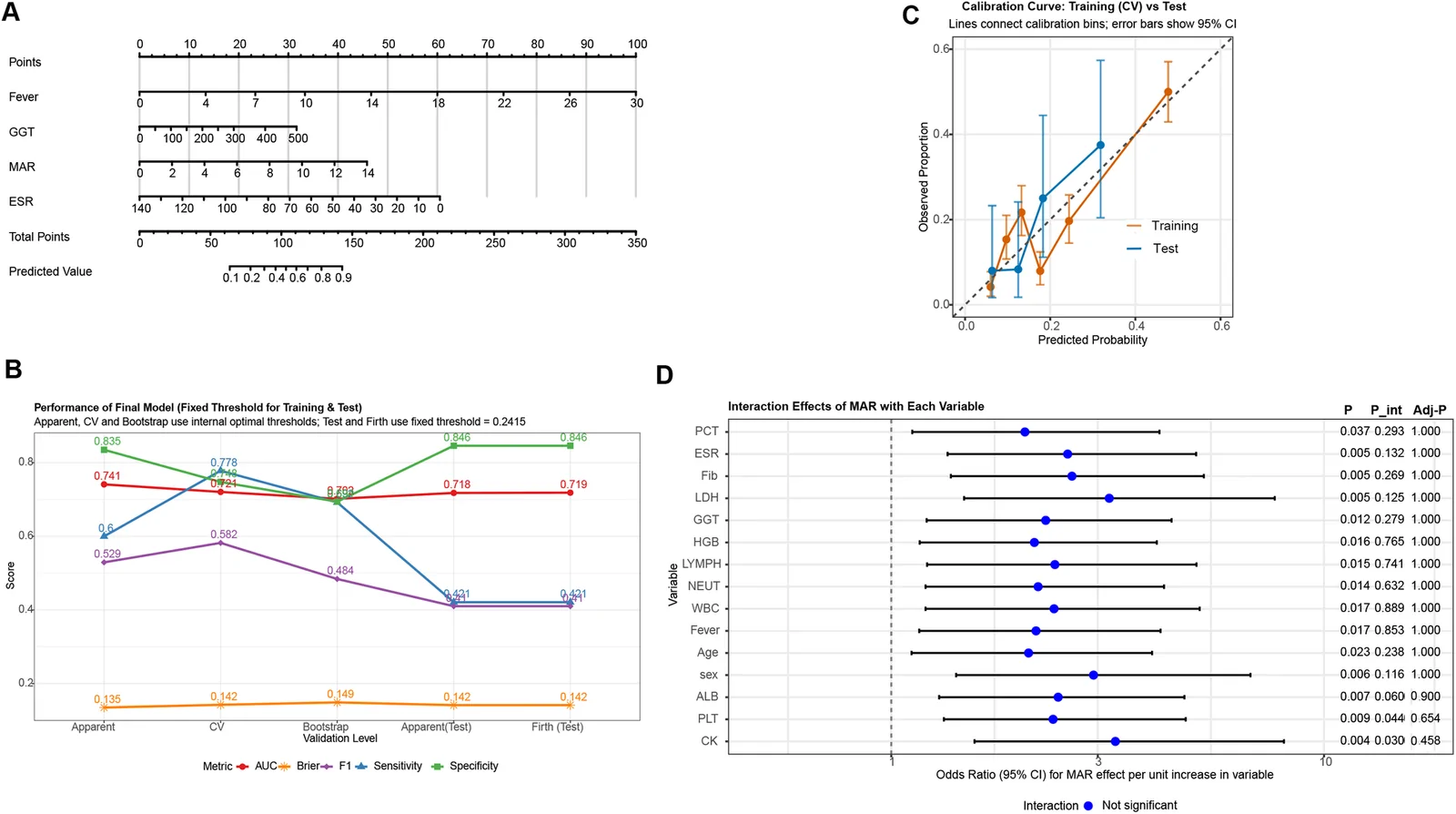
Nomogram, performance metrics, calibration curves, and interaction analysis of the final logistic regression model. **(A)** Nomogram for predicting baseline CAL risk. Points are assigned to each of the four independent predictors (fever duration, GGT, MAR, and ESR); the sum of the points corresponds to the predicted probability of CAL. **(B)** Summary of logistic regression performance metrics across different validation levels. Apparent, 10-fold repeated cross-validation, and bootstrap-corrected estimates are based on internal optimal thresholds; the split-sample test set and Firth penalized regression estimates are based on the fixed Youden index threshold of 0.2415 derived from the training set. Metrics shown include area under the ROC curve (AUC), Brier score, F1 score, sensitivity, and specificity. **(C)** Calibration curves for the final logistic model on the training set (based on 10-fold cross-validation) and the hold-out test set. The solid lines represent the observed proportions; the dashed line indicates ideal perfect calibration. Optimism-corrected calibration intercept and slope were −0.272 (95% CI: −0.546 to 0.007) and 0.791 (95% CI: 0.623 to 0.967). **(D)** Forest plot illustrating the interaction effects between MAR and each evaluated covariate. For each subgroup, the odds ratios (ORs) for MAR, raw *P* values, interaction *P* values (from likelihood ratio tests), and Bonferroni-corrected *P* values are shown. None of the interaction terms reached statistical significance after multiple testing correction (all adjusted *P* > 0.05).

Multivariable logistic regression identified four independent predictors for CAL: fever duration (OR = 1.20, 95% CI: 1.06–1.35, *P* 0.003), GGT (OR = 1.00, 95% CI: 1.00–1.01, *P* 0.018), MAR (OR = 1.19, 95% CI: 1.03–1.39, *P* 0.020), and ESR (OR = 0.97, 95% CI: 0.95–1.00, *P* 0.015). The final logistic regression equation is: logit(*P*) = −2.369 + 0.185 × (Fever duration) + 0.0035 × (GGT) + 0.181 × (MAR) − 0.024 × (ESR), where MAR = (monocyte count/albumin) × 100. Based on this model, a nomogram was constructed to facilitate individualized risk estimation in clinical practice (Figure 3A).

#### 3.3.2 Model performance and calibration

On the training set, the model showed an AUC of 0.741 (95% CI: 0.657–0.825), which dropped modestly after internal validation—0.721 (95% CI: 0.431–0.961) by repeated 10-fold cross-validation and 0.702 (95% CI: 0.661–0.736) by bootstrap correction. The wide cross-validation range likely reflects the small number of positive events (with CAL 19.8%). In the hold-out test set (*n* = 97), using the training-derived Youden threshold, the model achieved an AUC of 0.718 (95% CI: 0.584–0.852), consistent with the resampling estimates. Firth penalized regression gave nearly identical results (AUC = 0.719, 95% CI: 0.584–0.852), supporting model robustness. Of note, under this fixed threshold, sensitivity was low (0.421), meaning nearly 60% of CAL-positive patients would be missed, while specificity was high (0.846), suggesting the model performs better at ruling out CAL than detecting it-a trade-off that warrant threshold re-calibration for screening use (Figure 3B). Calibration was assessed using 10-fold CV and the test set (Figure 3C); the calibration curves closely followed the diagonal, with observed proportions within the 95% CIs of the binned predictions. The optimism-corrected calibration intercept was −0.272 (95% CI: −0.546 to 0.007), with the 95% CI containing the ideal value of 0, indicating no significant systematic bias. The calibration slope was 0.791 (95% CI: 0.623 to 0.967), with the upper bound falling slightly below the ideal value of 1, suggesting a mild tendency toward probability overdispersion. Additionally, the model achieved a Brier score of 0.142 on the test set, further supporting the absence of systematic miscalibration. It is important to emphasize that the hold-out test set AUC was derived from a single-center internal cohort. Given the limited number of CAL events in this test set (*n* = 19), these split-sample metrics should be interpreted descriptively and require cautious interpretation, rather than serving as confirmatory evidence.

#### 3.3.3 Supplementary analyses

Potential interactions between MAR and other clinical variables were explored (Figure 3D), but none reached statistical significance after Bonferroni correction (all adjusted *P* > 0.05). Finally, restricted cubic spline (RCS) regression with three knots was used to examine the dose–response relationship between MAR and CAL, adjusting for fever duration, ESR, and GGT (Supplementary Figure S1). The likelihood ratio test comparing the RCS model with a linear model yielded a *P* value of 0.750 for non-linearity, confirming that a linear term for MAR was appropriate in the final model.

### 3.4 Exploratory comparison of machine learning models and model selection

**Figure 4:**
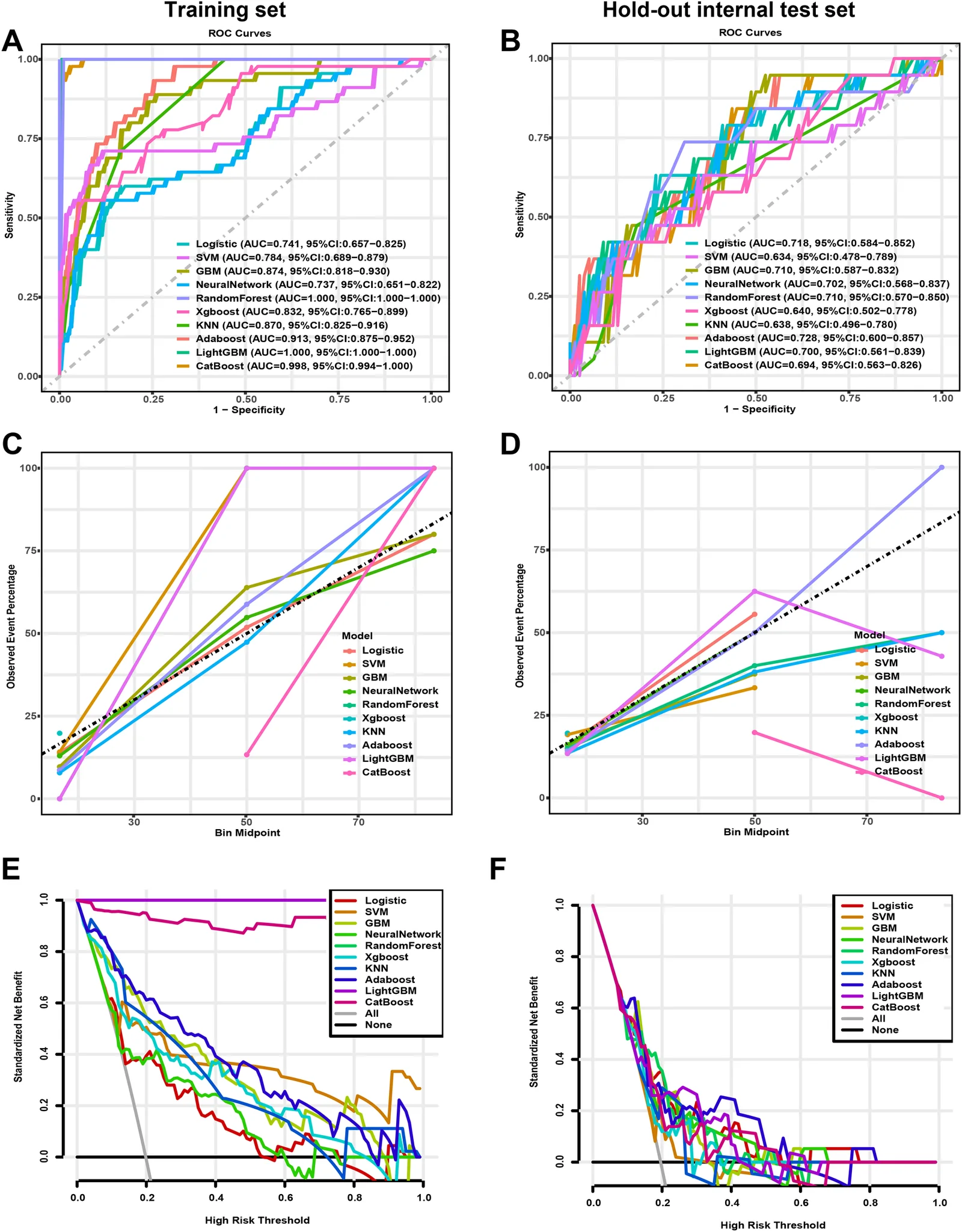
ROC, calibration curve, and DCA curves of the ten model. **(A)** ROC curve in the training set. **(B)** ROC curve in the hold-out internal test set. **(C)** Calibration curve in the training set. **(D)** Calibration curve in the hold-out internal test set. **(E)** DCA curve in the training set. **(F)** DCA curve in the hold-out internal test set. SVM, support vector machine; GBM, gradient boosting machine; NN, neural network; RF, random forest; XGBoost, extreme gradient boosting; KNN, k-nearest neighbors; DCA, decision curve analysis; ROC, receiver operating characteristic.

Ten machine learning algorithms were trained on the training set and evaluated on the hold-out internal test set using a fixed Youden-index threshold determined from the training set (Table 2). The overall classification performance and error distributions are summarized in Supplementary Figure S2, while discriminative ability, calibration, and net clinical benefit are depicted in Figure 4.

**Table 2:** Performance comparison of machine learning models on training and validation sets.

Model	Dataset	Threshold	Accuracy	Sensitivity	Specificity	Precision	F1	BrierScore
GBM	Training	0.1782	0.771	0.889	0.742	0.460	0.606	0.108
	Test	0.1782	0.608	0.684	0.590	0.289	0.406	0.147
SVM	Training	0.1775	0.850	0.711	0.885	0.604	0.653	0.128
	Test	0.1775	0.639	0.632	0.641	0.300	0.407	0.157
Logistic	Training	0.2415	0.789	0.600	0.835	0.474	0.529	0.135
	Test	0.2415	0.763	0.421	0.846	0.400	0.410	0.142
Adaboost	Training	0.1565	0.784	0.933	0.747	0.477	0.632	0.098
	Test	0.1565	0.629	0.579	0.641	0.282	0.379	0.139
Xgboost	Training	0.2055	0.731	0.778	0.72	0.407	0.534	0.147
	Test	0.2055	0.629	0.474	0.667	0.257	0.333	0.153
CatBoost	Training	0.5703	0.974	0.978	0.973	0.898	0.936	0.244
	Test	0.5703	0.722	0.421	0.795	0.333	0.372	0.272
NeuralNetwork	Training	0.2658	0.819	0.556	0.885	0.543	0.549	0.133
	Test	0.2658	0.794	0.316	0.910	0.462	0.375	0.144
RandomForest	Training	0.425	1.000	1.000	1.000	1.000	1.000	0.022
	Test	0.425	0.784	0.316	0.897	0.429	0.364	0.147
LightGBM	Training	0.4025	1.000	1.000	1.000	1.000	1.000	0.008
	Test	0.4025	0.794	0.316	0.91	0.462	0.375	0.153
KNN	Training	0.100	0.643	1.000	0.555	0.357	0.526	0.107
	Test	0.100	0.474	0.737	0.41	0.233	0.354	0.168

SVM, support vector machine; GBM, gradient boosting machine; KNN, k-nearest neighbors; XGBoost, extreme gradient boosting; LightGBM, light gradient boosting machine; CatBoost, categorical boosting.

Sensitivity = true positive rate, Specificity = true negative rate, Precision = positive predictive value, F1 score = harmonic mean of sensitivity and precision, Brier Score = mean squared difference between predicted probability and actual outcome (range 0–1; lower values indicate better probability calibration, with 0 representing perfect calibration). The threshold is the probability cut-off maximizing the Youden index (sensitivity + specificity − 1) on the training set, applied to both sets. Models marked with a training F1 score of 1.000 (Random Forest, LightGBM) show severe overfitting and should not be used for prediction. CatBoost also shows notably higher Brier scores, reflecting poor probability calibration.

**Figure 5:**
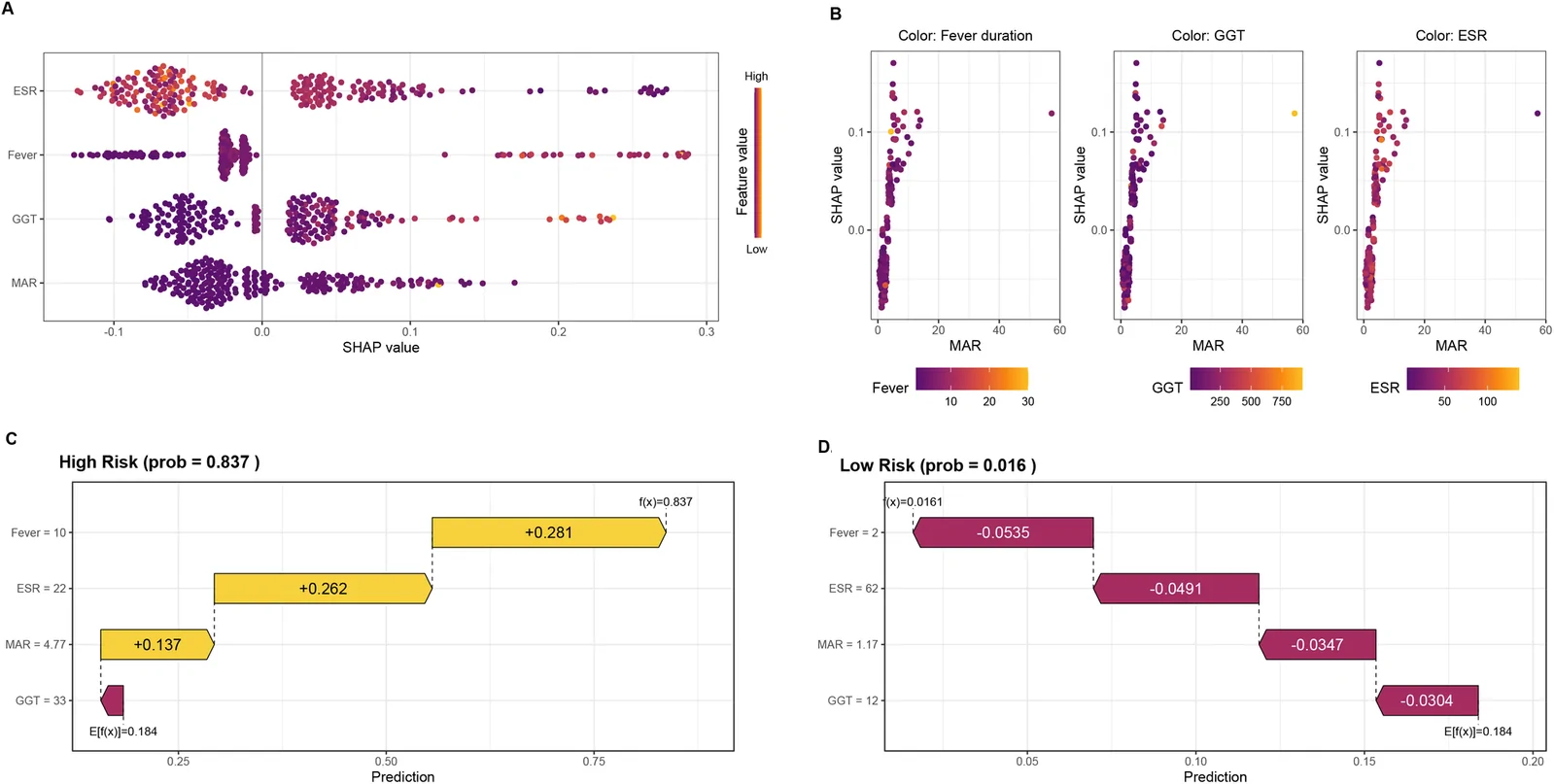
SHAP analysis of the GBM model. **(A)** SHAP summary plot showing the global impact of the four predictors. Points are colored by feature value. **(B)** SHAP dependence plots illustrating the interaction between MAR and fever duration (left), GGT (middle), and ESR (right). **(C,D)** Waterfall plots for representative high-risk (C; probability 0.837) and low-risk **(D)** probability 0.016) patients. Yellow and purple bars indicate positive and negative contributions to the log-odds, respectively; prolonged fever and elevated ESR were the dominant drivers in the high-risk case.

Among all models, logistic regression and GBM stood out as the most promising candidates. Logistic regression demonstrated consistent discriminative performance (training AUC 0.741 (95% CI: 0.657–0.825); test AUC 0.718 (95% CI: 0.584–0.852) (Figure 4), with a Brier score of 0.142 and high specificity (0.846), but limited sensitivity (0.421) on the test set. In contrast, GBM [training AUC 0.874 (95% CI: 0.818–0.930); test AUC 0.710 (95% CI: 0.587–0.832)] notably exhibited the highest sensitivity among non-overfitting models (0.684), outperforming logistic regression by 26.3 percentage points for CAL detection. This advantage, however, came at the expense of lower specificity (0.590) and a slightly higher Brier score (0.147). SHAP analysis of GBM model (Figures 5A,B) mechanistically revealed that elevated ESR and prolonged fever duration were the dominant drivers of high-risk assessments, and demonstrated positive synergistic interactions with MAR in driving model outputs. Figures 5C,5D illustrated contrasting risk profiles-a high-risk case (probability 0.837) vs. a low-risk case (probability 0.016).

**Figure 6:**
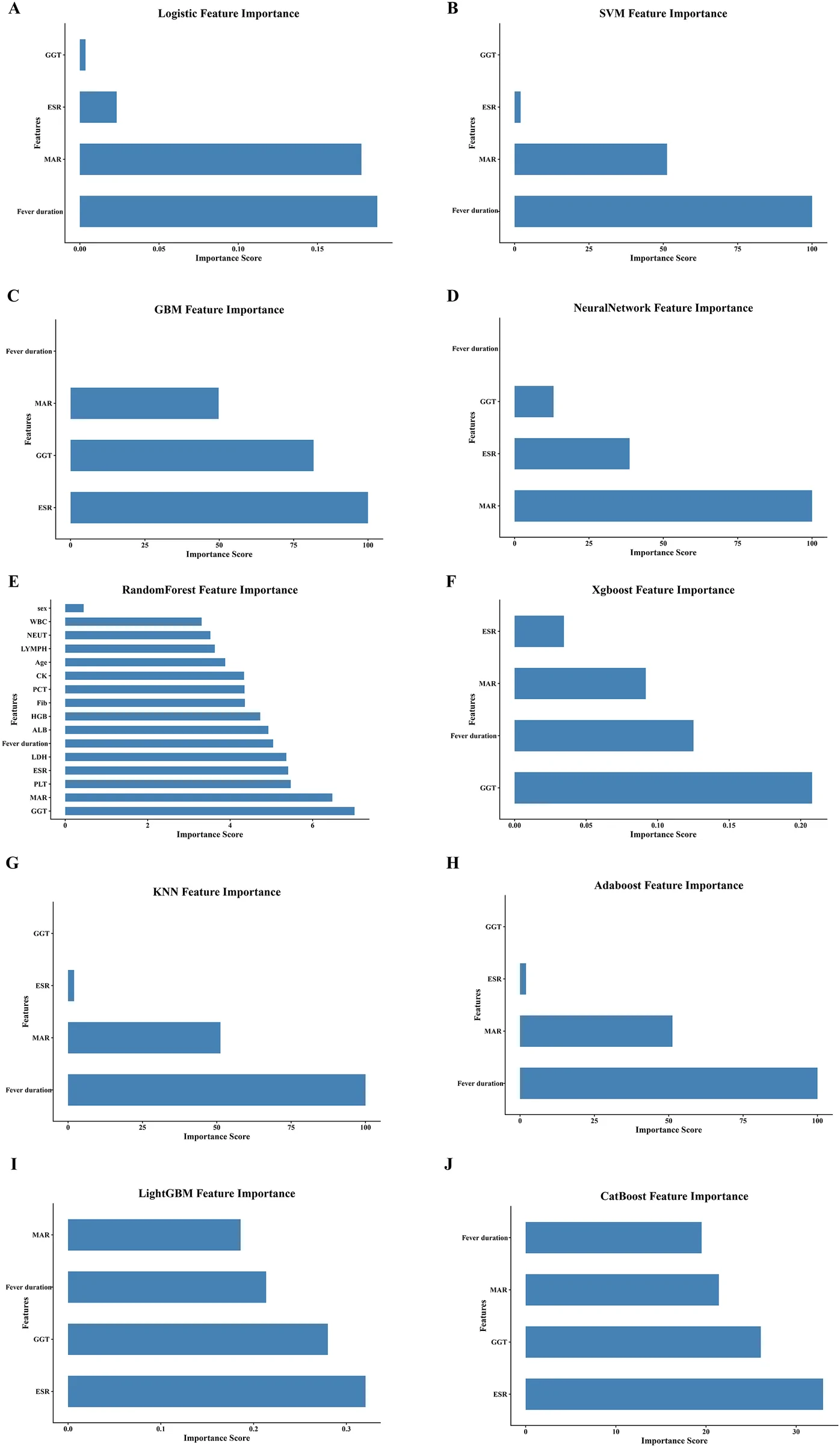
Variable importance plots for the ten machine learning models. Each panel shows the top three features ranked by importance (mean decrease in Gini for tree-based models, absolute coefficient or permutation importance for others).

Global feature importance (Figure 6) further reinforced MAR as a top-two predictor across most algorithms, supporting its clinical relevance. Regarding overfitting, Random Forest and LightGBM achieved perfect training performance (AUC 1.000) but collapsed on the test set (sensitivity 0.316). CatBoost also showed pronounced overfitting and poor calibration (test Brier 0.272). Crucially, these overfitted models are unequivocally unsuitable for clinical deployment, regardless of their training performance. Their inclusion in this study serves only as a negative benchmark to demonstrate that, for this modest dataset, overly complex algorithms do not guarantee generalizable utility and cannot be considered clinically meaningful. The remaining models (SVM, XGBoost, KNN, Neural Network, AdaBoost) underperformed relative to logistic and GBM, with test AUCs below 0.730 and inferior calibration (Table 2, Figure 4).

Taking together, logistic regression offers the best balance of specificity, calibration, and interpretability, making it a potentially interpretable option for scenarios where minimizing false positives is critical. Conversely, GBM's superior sensitivity may provide exploratory value in screening contexts, where reducing missed diagnoses is the priority. These findings suggest a potential conceptual combination between the two models-logistic regression maintaining robust specificity and GBM offering improved sensitivity-which, from an exploratory standpoint, could be conceptually combined to optimize early CAL risk assessment.

### 3.5 Online dynamic nomogram for exploratory use

A web-based dynamic logistic nomogram and GBM model were developed using the shiny package and deployed at https://liugaom.shinyapps.io/nomogram-kawasaki-CAL/. Clinicians can input fever duration before admission, GGT, ESR, monocyte count (MONO) and albumin (ALB)—the latter two are automatically converted to MAR = (MONO/ALB) × 100—to obtain an individualized CAL risk probability. Importantly, this web tool is strictly intended for exploratory research purposes only. The output must not be used for clinical decision-making, patient triage, or treatment guidance, as the underlying models are based on a single-center internal validation cohort and have not been independently validated externally. A prominent disclaimer has been embedded into the tool's interface to clearly state these limitations and emphasize the absolute requirement for multi-center external validation before any potential clinical translation.

## 4 Discussion

In this retrospective cohort of 324 children with KD, the incidence of CAL was 19.8%, which is similar to previously reported rates from East Asian populations. CAL represents the most serious long-term sequela of KD, with the potential to cause myocardial ischemia, acute coronary syndrome, heart failure, and sudden death in adulthood (24). Our relatively high CAL rate underscores the urgent need for simple, accurate tools to identify high-risk children early, before irreversible coronary damage develops. Currently, CAL risk assessment models for Kawasaki disease have evolved from traditional clinical scoring systems to multidimensional models based on machine learning and deep learning, incorporating emerging indicators such as hemodynamics, biomarkers, and genetic polymorphisms (25–27). However, clinical risk assessment models based on routine hematological tests remain cost-effective and offer advantages in resource-limited settings. Limited studies have explored novel composite indices that integrate different biological pathways. The MAR is a new index that captures both monocyte-driven innate immune activation and the negative acute phase response reflected by hypoalbuminemia. To our knowledge, no study has applied MAR to identify baseline CAL in KD. Our model incorporating MAR represents an initial step, but requires further validation before it can address this need.

We addressed these gaps by first using a consensus-based feature selection strategy (univariate, LASSO, random forest, Boruta, and RFE) to identify the most robust predictors. Fever duration, GGT, MAR, and ESR were consistently selected by all five methods and emerged as the final candidates. Then we compared ten machine learning algorithms. While complex models like GBM offered enhanced sensitivity for early screening, logistic regression was selected as the primary parsimonious model because it provided the best overall balance of discriminative performance, specificity, and clinical interpretability. Notably, GBM might offer additional sensitivity in exploratory screening contexts. The resulting nomogram and online calculator allow clinicians to input fever duration, GGT, ESR, and MAR—the latter automatically derived from routine monocyte and albumin measurements—to obtain an individual CAL risk probability, solely for exploratory research purposes.

The biological rationale for MAR is strongly supported by recent single-cell transcriptomic and mechanistic studies. Single-cell RNA sequencing of KD patients has consistently shown that monocytes are among the most differentially expressed cell populations during acute KD, with marked upregulation of pro-inflammatory mediators along with downregulation of MHC class II genes (28–30). Moreover, a CD14⁺CD16⁺ intermediate monocyte subset has been identified as a key player in IVIG responsiveness and coronary artery inflammation (31). In the acute phase, monocytes exhibit enhanced expression of the chemoattractant receptor CCR2 and produce high levels of monocyte chemoattractant protein-1 (MCP-1), which promotes further monocyte recruitment and vascular endothelial injury (32). Importantly, CD14⁺ monocytes act as central hubs in cell-cell communication during KD vasculitis, with SELPLG and ITK as important signaling genes (33). The accumulation of activated monocytes/macrophages in the coronary arterial wall is a hallmark histopathological finding of KD (34, 35). Albumin, on the other hand, is a negative acute-phase protein whose levels decrease in proportion to the severity of systemic inflammation. Hypoalbuminemia has been repeatedly identified as an independent risk factor for CAL (36, 37). Low albumin not only reflects impaired hepatic synthetic function but also indicates a state of poor nutritional reserve and reduced anti-oxidant capacity, which may compromise endothelial repair. Thus, MAR integrates two biologically distinct but synergistic signals: an elevated monocyte count (a direct measure of innate immune activation) and a reduced albumin level (a marker of heightened inflammation and impaired homeostatic reserve). An elevated MAR therefore reflects a “double-hit” scenario where active monocyte-mediated vascular inflammation coexists with diminished anti-inflammatory and repair capacity, jointly promoting CAL development. Consistent with this mechanistic understanding, our multivariable analysis confirmed that MAR, together with fever duration, GGT, and ESR, is independently associated with the presence of CAL. Sensitivity analyses further demonstrated the stability of the MAR coefficient, and restricted cubic spline analysis indicated a linear relationship, simplifying clinical application.

One unexpected finding was that ESR levels tended to be lower in the CAL group than in the non-CAL group, although the differences were not statistically significant (ESR *p* = 0.093). This contrasts with many previous studies that report higher acute-phase markers in CAL patients (38). Several explanations may account for this paradox. First, our CAL definition includes patients with mild transient coronary dilation, whose overall inflammatory burden might be less severe than those with persistent aneurysms. Second, the timing of blood sampling relative to the disease course could differ between groups; for example, CAL patients might have been sampled later in the febrile phase, when ESR and PCT begin to normalize. Third, age distribution or unnoticed antipyretic use might have influenced these markers. Therefore, when applying our model, clinicians should be aware that it is intended for use at baseline (before IVIG administration) and only for patients who meet our inclusion criteria.

The strengths of our study include the first introduction of MAR as a predictor for baseline CAL in KD, the systematic comparison of ten machine learning algorithms with a clear rationale for selecting a simple logistic model and exploring GBM as a potential complementary tool, and the use of a strict consensus-based procedure to enhance feature selection reliability. Moreover, rigorous sensitivity analyses (Firth regression and bootstrap) and an RCS test were performed to examine non-linearity, which together support the reliability of our findings. The reported optimism-corrected calibration intercept (−0.272, 95% CI: −0.546 to 0.007) and slope (0.791, 95% CI: 0.623 to 0.967) provided further evidence of acceptable calibration. Several limitations must be acknowledged. First, the statistical variability introduced by multiple imputation is not fully captured by the pooling of five MICE imputations into a single median dataset for all downstream modeling steps in this study. However, given the exploratory nature of this study and the need for a reproducible and computationally feasible workflow for internal model development, this pragmatic pooling approach was considered appropriate and transparent for the present analysis. While the available CAL events in the training set (*n* = 45) were calculated to yield an EPV of 11.25, which satisfies the EPV >10 criterion for our four-predictor model, this modest number precluded inclusion of additional predictors and limited power for reliable subgroup testing (e.g., the significant LDH interaction may represent a chance finding). Moreover, the split hold-out internal test set contained only 19 CAL events, rendering its performance metrics unstable; therefore, our internal validation primarily relied on bootstrap-corrected estimates. The multi-algorithm comparisons were exploratory, and the severe overfitting of complex models justified our deliberate choice of parsimonious logistic regression. Critically, all validation performed here is internal only-the hold-out set was from the same single center-and bootstrap resampling, while mitigating overfitting, cannot account for between-center differences in patient demographics, disease severity, or treatment protocols. Importantly, the generalizability of our proposed model to other populations, ethnic groups, or healthcare settings remains uncertain. Variations in baseline patient characteristics, local diagnostic criteria, clinical practices, and IVIG administration protocols across different centers could substantially alter the model's discriminative performance and calibration. Therefore, the current findings should not be interpreted as evidence of broad applicability. Given this single-center Chinese cohort, generalizability to other populations is uncertain, and dynamic MAR changes were not assessed. Consequently, all results are strictly hypothesis-generating; external validation in independent, prospective multicenter cohorts is mandatory before any clinical implementation. Until then, our online calculator should serve only as a low-cost research tool for exploratory risk assessment.

## 5 Conclusions

In conclusion, we have developed a simple, interpretable logistic regression model based on fever duration, GGT, ESR, and the novel MAR for detecting baseline CAL in children with KD. Although GBM might provide additional sensitivity in exploratory screening scenarios, the logistic model may be considered when specificity is prioritized. MAR, supported by mechanistic evidence, may serve as a potential biomarker for CAL risk, but prospective multi-center studies are essential to validate and refine this tool. However, given the limited number of CAL events and its single-center origin, all analyses should be considered strictly exploratory. The model's generalizability to broader populations is unknown; multi-center external validation is an absolute prerequisite before any clinical application or clinical decision-making.

## Data Availability

The original contributions presented in the study are included in the article/Supplementary Material, further inquiries can be directed to the corresponding author/s.

## Supplementary material

Supplementary Figure S1 Restricted cubic spline (RCS) analysis of MAR.

Supplementary Figure S2 Confusion matrices of the ten machine learning models in the training and hold-out internal test sets. For each model, the matrix shows the number and percentage of correctly and incorrectly classified CAL (yes/no) cases. Rows represent true labels, columns represent predictions. The color intensity reflects the proportion of cases in each cell.
