# Supplementary material for: Development and internal validation of a monocyte-to-albumin ratio-based risk assessment model for coronary artery lesions in Kawasaki disease
Source: Front Pediatr. 2026 Aug 18;14:1881684. doi: 10.3389/fped.2026.1881684 (PMC13529742; doi:10.3389/fped.2026.1881684)
Supplement: Supplementary file 1 [file Table1.docx]

| **Supplementary Table 1 Baseline characteristics of the patients in training set and validation set.** | | | | |
| --- | --- | --- | --- | --- |
|  | Overall | Training | Test | p |
|  |  | 227 | 97 |  |
| CAL | No | 182 (80.2) | 78 (80.4) | 1.000 |
|  | Yes | 45 (19.8) | 19 (19.6) |  |
| Sex (%) | Male | 149 (65.6) | 61 (62.9) | 0.703 |
|  | Female | 78 (34.4) | 36 (37.1) |  |
| Age (months) |  | 24.00 [12.00, 48.00] | 24.00 [12.00, 48.00] | 0.633 |
| Fever duration (days) |  | 5.00 [4.00, 6.00] | 5.00 [4.00, 6.00] | 0.711 |
| WBC count (*109/L) |  | 15.30 [12.10, 18.90] | 15.20 [11.10, 20.30] | 0.986 |
| NEUT count (*109/L) |  | 72.20 [59.35, 81.50] | 70.30 [62.60, 80.60] | 0.720 |
| LYMPH count (*109/L) |  | 2.78 [1.73, 4.56] | 2.69 [1.87, 3.98] | 0.721 |
| PLT (*109/L) |  | 338.00 [273.50, 427.00] | 324.00 [262.00, 399.00] | 0.251 |
| HGB (g/L) |  | 114.00 [106.00, 123.00] | 115.00 [103.00, 123.00] | 0.784 |
| ALB (g/L) |  | 35.60 [32.98, 38.00] | 35.45 [32.50, 38.10] | 0.614 |
| ALB missing | 4 (1.2%) | 3 (1.3%) | 1 (1.0%) |  |
| GGT (U/L) |  | 55.00 [18.00, 157.00] | 45.00 [17.00, 137.00] | 0.429 |
| GGT missing | 2 (0.6%) | 2 (0.9%) | 0 (0.0%) |  |
| CK (U/L) |  | 49.00 [31.00, 75.00] | 47.00 [29.00, 74.00] | 0.395 |
| CK_missing | 4 (1.2%) | 4 (1.8%) | 0 (0.0%) |  |
| LDH (U/L) |  | 304.00 [261.50, 358.50] | 295.00 [247.00, 343.00] | 0.243 |
| LDH_missing | 4 (1.2%) | 4 (1.8%) | 0 (0.0%) |  |
| Fib (g/L) |  | 6.01 [5.17, 6.83] | 6.03 [5.28, 7.01] | 0.702 |
| Fib_missing | 27 (8.3%) | 20 (8.8%) | 7 (7.2%) |  |
| ESR (mm/h) |  | 47.00 [34.00, 62.00] | 51.00 [40.00, 62.00] | 0.321 |
| ESR_missing | 11 (3.4%) | 7 (3.1%) | 4 (4.1%) |  |
| PCT (ng/mL) |  | 0.82 [0.30, 2.24] | 0.78 [0.36, 2.36] | 0.774 |
| PCT_missing | 2 (0.6%) | 1 (0.4%) | 1 (1.0%) |  |
| MAR |  | 2.57 [1.73, 3.78] | 2.52 [1.68, 3.94] | 0.705 |
| MAR missing | 4 (1.2%) | 3 (1.3%) | 1 (1.0%) |  |

**Abbreviations:** CAL, coronary artery lesions; WBC, white blood cell count; NEUT, absolute neutrophil count; LYMPH, absolute lymphocyte count; PLT, platelet count; HGB, hemoglobin; ALB, albumin; GGT, gamma‑glutamyl transferase; CK, creatine kinase; LDH, lactate dehydrogenase; Fib, fibrinogen; ESR, erythrocyte sedimentation rate; PCT, procalcitonin; MAR, monocyte‑to‑albumin ratio (calculated as monocyte count/albumin × 100).
